# Knowledge and Acceptability of Human Papillomavirus Vaccination among Women Attending the Gynaecological Outpatient Clinics of a University Teaching Hospital in Lagos, Nigeria

**DOI:** 10.1155/2017/8586459

**Published:** 2017-12-19

**Authors:** Kehinde S. Okunade, Oyebola Sunmonu, Gbemisola E. Osanyin, Ayodeji A. Oluwole

**Affiliations:** ^1^Department of Obstetrics & Gynaecology, College of Medicine, University of Lagos, Lagos, Nigeria; ^2^Department of Obstetrics & Gynaecology, Lagos University Teaching Hospital, Lagos, Nigeria

## Abstract

**Objectives:**

This study was aimed at determining the knowledge and acceptability of HPV vaccine among women attending the gynaecology clinics of the Lagos University Teaching Hospital (LUTH).

**Methods:**

This was a descriptive cross-sectional study involving 148 consecutively selected women attending the gynaecology clinic of LUTH. Relevant information was obtained from these women using an interviewer-administered questionnaire. The data was analysed and then presented by simple descriptive statistics using tables and charts. Chi-square statistics were used to test the association between the sociodemographical variables and acceptance of HPV vaccination. All significance values were reported at *P* < 0.05.

**Results:**

The mean age of the respondents was 35.7 ± 9.7 years. The study showed that 36.5% of the respondents had heard about HPV infection while only 18.9% had knowledge about the existence of HPV vaccines. Overall, 81.8% of the respondents accepted that the vaccines could be administered to their teenage girls with the level of education of the mothers being the major determinant of their acceptability (*P* = 0.013).

**Conclusions:**

Awareness of HPV infections and existence of HPV vaccines is low. However, the acceptance of HPV vaccines is generally high. Efforts should be made to increase the awareness about cervical cancer, its aetiologies, and prevention via HPV vaccination.

## 1. Introduction

Cervical cancer is the most common gynaecological cancer among women in sub-Saharan Africa and a leading cause of cancer deaths in Nigeria [1]. In 2012 there were an estimated 528,000 new cases of cervical cancer and 266,000 deaths from cervical cancer, with 70% of those deaths occurring in developing countries [2]. The annual number of cervical cancer cases in Nigeria is 14,089 and the annual number of deaths is 8,240 [3]. This is a major public health challenge and the number may increase if efforts toward its reduction are not put in place.

Various molecular and epidemiological evidences have shown that cervical cancer is caused by oncogenic human papillomavirus (HPV), a sexually transmitted infection, especially serotypes 16, 18, and 31 [4–6]. HPV are small DNA viruses, of which there are several types associated with benign and malignant conditions of the anogenital areas and oropharynx [7, 8]. These viruses are ubiquitous and most women in the world are probably infected with at least one type of HPV during their sexual life giving a point prevalence of 10.1% [8]. Currently, it is estimated that about 23.7% of women in the general population in Nigeria harbour cervical HPV infection at a time [3].

One of the major prevention strategies for cervical cancer is vaccination against HPV infection among adolescents prior to their first sexual exposure [9]. The approval and recommendation of two HPV vaccines (Gardasil and Cervarix) has now provided a huge opportunity to curb the burden of cervical cancer [5, 9]. Providing HPV vaccines in low- and middle-income countries is a critical pillar for meeting the global action plan for closing the cancer divide [10]. The World Health Organisation (WHO) recommends offering HPV vaccine to girls at the age of 9 to 13 years, prior to sexual exposure, since the vaccine has the highest efficacy if girls have not already acquired HPV infection [11]. The Nigerian Federal Ministry of Health (FMOH) recommends that immunization against HPV be around ages 9 to 26 years and usually before the start of sexual activity [12]; however, unlike the vaccines for childhood diseases, HPV vaccination in Nigeria is not currently provided during routine or free mass immunization programmes, but only available through “out-of-pocket” purchase for individual use [13].

Fortunately, in 2013, the vaccine manufacturers (Merck and GSK) offered the Global Alliance on Vaccines and Immunization (GAVI) a reduced price of US$4.50 per dose from the initial selling price of US$30–40 [14]. This reduction was made in order to ensure access for developing countries, which need the vaccine the most. However, for GAVI new vaccines support, only countries with three doses of diphtheria, tetanus, and pertussis vaccines (DTP3) coverage levels greater than or equal to 70% may apply. Nevertheless, countries that have not attained DTP3 coverage levels of up to 70% may apply to GAVI for HPV demo. The HPV demo means that poor countries that have not yet established the ability to deliver HPV vaccine could be supported by GAVI to implement a two-year demonstration programme. Nigeria, with DTP3 coverage of 41%, was only eligible for the HPV demo programme between 2013 and 2015 [14].

Despite these efforts, barriers to obtaining these vaccines individually in low-income countries like Nigeria still remain as the cost of the vaccine is prohibitive and out of the reach of the poor. Other major contributors to this are the poor vaccine delivery efforts, low cervical cancer screening levels, ineffective health system capabilities, inaccessibility to medical care, low awareness and knowledge of HPV and cervical cancer, and failure to recognize cervical cancer as a major health concern [10, 13, 15–18]. This study, therefore, was aimed at finding out the level of knowledge about these HPV vaccines among mothers and their acceptance of the vaccines for their adolescent girls since the GAVI new vaccines support efforts of 2013 to 2015.

## 2. Materials and Methods

### 2.1. Study Design and Setting

This study was a descriptive cross‐sectional survey that involved women attending the gynaecology outpatient clinics of the Lagos University Teaching Hospital (LUTH) between August and October 2015. LUTH is the teaching hospital of the College of Medicine of the University of Lagos. It is situated in the heart of Lagos Mainland, which has a population of over 2,150,100 inhabitants. It serves the whole of Lagos and its suburbs, and it is open to all categories of patients. The hospital has over 800-bed spaces and records over 9,000 patients' attendances per month. It operates at the tertiary level of the healthcare delivery system in Nigeria. Referrals are received from both private and public hospitals in Lagos as well as other parts of the country.

### 2.2. Study Population

These were nonpregnant women with non-life threatening gynaecological complaints attending the gynaecological outpatient and cytology clinics of LUTH during the study period and the sample population included all consenting women recruited by consecutive sampling method. Excluded from the study were women being evaluated or managed for any gynaecological malignancy.

### 2.3. Data Collection

Informed consent was obtained from the women prior to their recruitment for the study and interviews were conducted by the investigators to collect relevant data from the participants. The interview guide was divided into three main sections (A to C) which included data on (A) sociodemographic variables of participating women, (B) their knowledge of genital HPV infection and HPV vaccines, and (C) their acceptance of HPV vaccines for their adolescent girls after 5- to 10-minute group health talks on HPV vaccination.

### 2.4. Statistical Analysis

Relevant data were analysed using SPSS statistical package version 21.0 for windows manufactured by IBM Corp., Armonk, NY, United States. The data were presented as chart and frequency distribution tables. The association between the sociodemographic variables (age and educational level) and acceptance of HPV vaccination was tested using Chi-square test or Fisher's exact test where appropriate. All significance values were reported at *P* < 0.05.

### 2.5. Ethical Approval

Ethical approval for the study (HREC Number:* ADM/DCST/HREC/2179*) was obtained from the hospital's Health Research and Ethics Committee prior to the commencement of the study and the ethical principles according to the Helsinki Declaration were considered during the course of the research.

## 3. Results

Out of the 151 questionnaires administered during the study, 148 were properly filled, giving a response rate of 98.0%. The mean age of the respondents was 35.7 ± 9.74 with an age range of 20–65 years. Majority of the respondents (41.9%) were between 26 and 35 years. The largest proportion of the respondents were married (68.9%), had one or more daughters (58.1%), and had at least tertiary level of education (82.4%) (Table 1).

**Figure 1:**
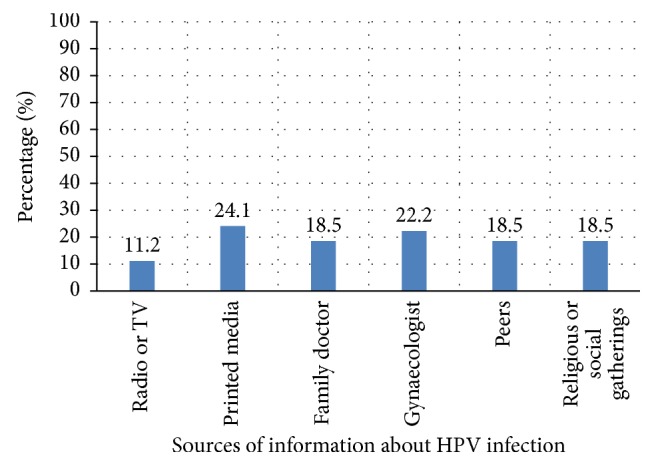
Reported sources of information among respondents with correct knowledge of HPV infection (*n* = 43) (*P* > 0.091).

Only 54 (36.5%) of the respondents have heard about genital HPV infection with a large proportion of them (87.0%) knowing correctly that it was a sexually transmitted infection while a further 91.5% of these knew that it can cause cervical cancer (Table 2) thus giving a generally good knowledge of 29.1% (43/148) of HPV infection and its association with cervical cancer. A major proportion of these 43 respondents (24.1%) got their information from the print media although there were no statistically significant differences between all the different sources of information (*P* > 0.091) (Figure 1). However, only 28 of these 43 respondents (65.1%) which is equivalent to 18.9% of the total study participants were aware of the existence of the HPV vaccines (Table 3). Although the respondents' age (*P* = 0.098) and them having a daughter (*P* = 0.084) did not influence their awareness of the existence of HPV vaccination, their level of education played a significant role in this respect (*P* = 0.001) (Table 3). Three of the 43 very knowledgeable respondents (6.9%) have had the HPV vaccines administered to them personally in the past. All these 3 women were between 36 and 40 years of age, each with at least tertiary level of education, and they all had the vaccine at private health facilities in Lagos.

A major proportion of the total respondents (81.8%) expressed their willingness to vaccinate their daughters while the others were either unwilling (10.8%) or indifferent (7.4%) to the idea of vaccination against HPV infection (Table 4). The reasons given by those respondents who were unwilling or indifferent to vaccinating their daughters were the high cost (55.6%), concerns about the side effects (48.1%), and poor availability (25.9%) of the vaccines. A good number of these respondents (14.8%) could not give any specific reason for their lack of willingness to accept this vaccination.

There was a statistically significant association between the respondents' level of education and their willingness to accept HPV vaccination for their daughters (*P* = 0.013) but no similar associations were observed with the respondents' age (*P* = 0.256) and them having a daughter (*P* = 0.101) (Table 5), although there is a trend for age as knowledge of HPV vaccination and the willingness to accept the vaccine among the respondents decrease progressively with increasing age (Tables 3 and 5).

## 4. Discussion

This study reported a very low awareness level of HPV infection (36.5%) despite a high literacy level of over 80% with tertiary education among the respondents. This finding is somehow similar to the report from a community-based study carried out in Shomolu local government area of Lagos State where a similarly low level of awareness (27.9%) was reported [17]. Similar results were also found in previous studies carried out within [15, 19] and outside Nigeria [20, 21]. However, in contrast to these, other studies carried out among health workers in Lagos [18], Enugu [22], and South Africa [23] showed a relatively high knowledge of 85.0%, 74.0%, and 96.0%, respectively. The disparity in awareness in these latter studies is likely due to the higher exposure of these health workers to information about HPV infection in the hospital. The different sources of information about HPV infection as reported in this study also showed that if properly utilised, information about HPV infection and cervical cancer can be passed through different fora thus ensuring generally wide population coverage.

This current study just like other previous studies carried out in Nigeria with awareness of HPV vaccines ranging between 19.7% and 25.3% [16–18] had also shown a similarly low level of awareness of 18.9%. This contrasted sharply with other studies done in Kenya [24] and South Africa [23] where generally high-level knowledge of HPV vaccination had been reported. This could be attributed to the introduction of the routine HPV vaccination programme through the expanded programme on immunization (EPI) and the school health systems in these other countries unlike in Nigeria where such government-initiated programmes are still largely unavailable.

The high level of willingness shown by the respondents in this study (81.8%) to accept HPV vaccinations for their daughters is consistent with the reports from previous studies in Nigeria with levels of acceptance ranging between 70.0% and 88.9% [16–18, 25] and also similar to those conducted in Tanzania (93.0%) [26], South Africa (89.0%) [23], Singapore (87.1%) [27], and Honduras (91.0%) [28] in which high levels of willingness to recommend the vaccine to others or to accept it for themselves were shown by majority of the respondents. This indicated that, despite the low knowledge about the vaccine, most women if given the necessary information and education will be very willing to get vaccinated or recommend the vaccine to their loved ones.

The finding of a significantly higher level of knowledge and acceptance of HPV vaccination among women with higher educational qualifications in this study was a likely indication that the more educated women are generally more exposed to information on HPV infection and vaccination against it from various sources just as previously reported in various other studies [16, 19, 20, 22, 24]. The major limitation to this study was that it was totally hospital-based and the findings may not be representative of the general population. The high level of awareness and willingness to accept vaccination may actually be skewed as a result of the relatively high educational level of the vast majority of the respondents recruited for the study compared to the general population of Lagos women.

## 5. Conclusion

The low awareness of the existence and availability of the HPV vaccine in this study showed that much work is still needed to create awareness for HPV vaccines in Nigeria. This can be achieved with the development of health promotion and educational strategies for the public considering that education was shown to have significantly influenced the level of acceptance of the vaccines. It is therefore recommended that continuous efforts should be made to improve mothers' knowledge through education and to also ensure the possible inclusion of the vaccine in the national immunization schedule.

## Figures and Tables

**Table 1:** Sociodemographic characteristics of respondents (*n* = 148).

Variable	Frequency	%
*Age (in years)*		
<25	20	13.5
26–35	62	41.9
36–45	40	27.0
>45	26	17.6
*Mean ± SD* = 35.7 ± 9.74	*Age range* = 20–65 years	
*Existence of one or more daughters*		
No daughter	62	41.9
One or more daughters	86	58.1
*Marital status*		
Single	37	25.0
Married	102	68.9
Others	9	6.1
*Level of education*		
Uneducated	1	0.7
Primary	5	3.4
Secondary	20	13.5
Tertiary	122	82.4

**Table 2:** Knowledge of HPV Infection.

Knowledge	Frequency	%
*Having ever heard of HPV infection *(*n* = 148)		
Yes	54	36.5
No	94	63.5
*Mode of transmission of genital HPV infection *(*n* = 54)		
Sexually transmitted	47	87.0
Ingestion of contaminated food	2	3.7
Drinking of contaminated water	0	0.0
Kissing	5	9.3
*HPV infection causing cervical cancer *(*n* = 47)		
Yes	43	91.5
No	4	8.5

**Table 3:** Association between sociodemographic characteristics and awareness of HPV vaccination among respondents with correct knowledge of HPV infection (*n* = 43).

Characteristics	*N*	Awareness of HPV vaccination		*P* value
		Aware (%)	Not aware (%)	
*Age of respondents (in years)*				0.098^F^
<25	5	4 (80.0)	1 (20.0)	
26–35	20	15 (75.0)	5 (25.0)	
36–45	16	9 (56.2)	7 (43.8)	
>45	2	0 (0.0)	2 (100.0)	
*Mean ± SD*		31.3 ± 5.7	37.9 ± 11.6	
*Existence of one or more daughters*				0.084
No daughter	14	9 (64.3)	6 (35.7)	
One or more daughters	29	19 (65.5)	9 (34.5)	
*Level of education*				0.001
Less than tertiary	11	1 (9.1)	10 (90.9)	
At least tertiary	32	27 (84.4)	5 (15.6)	

*Total*	*43*	*28 (65.1)*	*15 (34.9)*	

*Note*. F: association determined by Fisher's exact test.

**Table 4:** Acceptance of HPV vaccination by respondents.

HPV vaccination	Frequency	%
*Willingness to vaccinate daughters *(*n* = 148)		
Yes	121	81.8
No	16	10.8
Indifferent	11	7.4
*Reasons for lack of willingness to vaccinate daughters *(*n* = 27)^*∗*^		
Costly	15	55.6
Nonavailability	7	25.9
Fear of side effects	13	48.1
No specific reason	4	14.8

^**∗**^Multiple options.

**Table 5:** Association between sociodemographic characteristics of respondents and their acceptance of HPV vaccination for their adolescent girls (*N* = 148).

Characteristics	*N*	Acceptance of HPV vaccination		*P* value
		Accepted (%)	Not accepted (%)	
*Age of respondents (in years)*				0.256
<25	20	17 (85.0)	3 (15.0)	
26–35	62	52 (83.9)	10 (16.1)	
36–45	40	32 (80.0)	8 (20.0)	
>45	26	20 (76.9)	6 (13.1)	
*Mean ± SD*		37.4 ± 9.7	39.8 ± 5.3	
*Existence of one or more daughters*				0.101
No daughter	62	49 (79.0)	13 (21.0)	
One or more daughters	86	72 (83.7)	14 (16.3)	
*Level of education*				0.013
Less than tertiary	25	17 (68.0)	8 (32.0)	
At least tertiary	123	104 (84.5)	19 (15.5)	

*Total*	*148*	*121 (81.8)*	*27 (18.2)*	

## References

[1] Thomas J., Ojemakinde O., Izebvaye I. (2002). Current concepts in cervical carcino-genesis and new perspectives in prevention. *Archives of Ibadan Medicine*.

[2] Ferlay J., Shin H. R., Bray F., Forman D., Mathers C., Parkin D. M. (2010). Estimates of worldwide burden of cancer in 2008: GLOBOCAN 2008. *International Journal of Cancer*.

[3] WHO/ICO (2014). Human Papilloma Virus and Related Diseases Report - Nigeria.

[4] Prat J. (2012). Pathology of cancers of the female genital tract. *International Journal of Gynecology and Obstetrics*.

[5] Erickson B. K., Alvarez R. D., Huh W. K. (2013). Human papillomavirus: What every provider should know. *American Journal of Obstetrics & Gynecology*.

[6] Patanwala I. Y., Bauer H. M., Miyamoto J., Park I. U., Huchko M. J., Smith-Mccune K. K. (2013). A systematic review of randomized trials assessing human papillomavirus testing in cervical cancer screening. *American Journal of Obstetrics & Gynecology*.

[7] Formana D., de Martel C., Lacey C. J. (2012). Global burden of human papillomavirus and related diseases. *Vaccine*.

[8] Akarolo-Anthony S. N., Famooto A. O., Dareng E. O. (2014). Age-specific prevalence of human papilloma virus infection among Nigerian women. *BMC Public Health*.

[9] Human Papillomavirus Vaccination (2014). The American College of Obstetricians and Gynaecologists Committee Opinion no. 588. *Obstetrics & Gynecology*.

[10] Perlman S., Wamai R. G., Bain P. A., Welty T., Welty E., Ogembo J. G. (2014). Knowledge and awareness of HPV vaccine and acceptability to vaccinate in sub-Saharan Africa: A systematic review. *PLoS ONE*.

[11] WHO (2009). World Health Organization Human papillomavirus vaccines WHO position paper. *The Weekly Epidemiological Record*.

[12] Federal Ministry of Health (2008). *Nigeria Cancer Control Plan 2008-2013*.

[13] Ajah L. O., Iyoke C. A., Ezeonu P. O., Ugwu G. O., Onoh R. C., Ibo C. C. (2015). Association between Knowledge of Cervical Cancer/Screening and Attitude of Teachers to Immunization of Adolescent Girls with Human Papilloma Virus Vaccine in Abakaliki, Nigeria. *American Journal of Cancer Prevention*.

[14] Preventing cancer: The role of vaccines. The Committee Encouraging Corporate Philanthropy in Nigeria. http://www.cecpng.org/.

[15] Agida T., Akaba G., Isah A., Ekele B. (2015). Knowledge and perception of human papilloma virus vaccine among the antenatal women in a Nigerian tertiary hospital. *Nigerian Medical Journal*.

[16] Odetola T. D., Ekpo K. (2012). Nigerian women's perceptions about human papilloma virus immunisations. *Journal of Community Medicine & Health Education*.

[17] Ezenwa B. N., Balogun M. R., Okafor I. P. (2013). Mothers' human papilloma virus knowledge and willingness to vaccinate their adolescent daughters in Lagos, Nigeria. *International Journal of Women's Health*.

[18] Makwe C. C., Anorlu R. I. (2011). Knowledge of and attitude toward human papillomavirus infection and vaccines among female nurses at a tertiary hospital in Nigeria. *International Journal of Women's Health*.

[19] Akanbi O. A., Iyanda A., Osundare F., Opaleye O. O. (2015). Perceptions of Nigerian Women about Human Papilloma Virus, Cervical Cancer, and HPV Vaccine. *Scientifica*.

[20] Poole D. N., Tracy J. K., Levitz L. (2013). A cross-sectional study to assess HPV knowledge and HPV vaccine acceptability in Mali. *PLoS ONE*.

[21] Montgomery M. P., Dune T., Shetty P. K., Shetty A. K. (2015). Knowledge and Acceptability of Human Papillomavirus Vaccination and Cervical Cancer Screening among Women in Karnataka, India. *Journal of Cancer Education*.

[22] Ojiyi C. E., Dike E. I., Okeudo C., Nzewuihe A. C., Uzoma M. J. K. (2013). Human papillomavirus vaccine: Awareness and acceptability amongst female medical students and health workers in a University Teaching Hospital in Eastern Nigeria. *Nigerian Journal of Surgical Sciences*.

[23] Hoque M. E. (2015). Acceptability of human papillomavirus vaccination among academics at the University of KwaZulu-Natal, South Africa. *South African Family Practice*.

[24] Masika M. M., Ogembo J. G., Chabeda S. V., Wamai R. G., Mugo N. (2015). Knowledge on HPV vaccine and cervical cancer facilitates vaccine acceptability among school teachers in Kitui County, Kenya. *PLoS ONE*.

[25] Ezeanochie M. C., Olagbuji B. N. (2014). Human papilloma virus vaccine: determinants of acceptability by mothers for adolescents in Nigeria. *African Journal of Reproductive Health*.

[26] Cunningham M. S., Skrastins E., Fitzpatrick R. (2015). Cervical cancer screening and HPV vaccine acceptability among rural and urban women in Kilimanjaro Region, Tanzania. *BMJ Open*.

[27] Ezat S. W. P., Hod R., Mustafa J., Dali A. Z. H. M., Sulaiman A. S., Azman A. (2013). National HPV immunisation programme: Knowledge and acceptance of mothers attending an obstetrics clinic at a teaching hospital, Kuala Lumpur. *Asian Pacific Journal of Cancer Prevention*.

[28] Perkins R. B., Langrish S. M., Cotton D. J., Simon C. J. (2011). Maternal support for human papillomavirus vaccination in honduras. *Journal of Women's Health*.

